# Gp350-targeted CAR-T therapy in EBV-positive Burkitt lymphoma: pre-clinical development of gp350 CAR-T

**DOI:** 10.1186/s12967-025-06188-w

**Published:** 2025-02-10

**Authors:** Jiajia Wang, Huiping Wang, Yangyang Ding, Nengneng Cao, Fengya Nan, Fan Wu, Cong Li, Xue Liang, Meng Xiao, Jinjing Guo, Zhimai Gao, Li Yan, Tielin Zhou, Yanli Li, Zhimin Zhai

**Affiliations:** 1 Department of Hematology/Hematologic Diseases Research Center, The Second Affiliated Hospital of Anhui Medical University, Hefei, 230601 Anhui China; 2 Department of Pathology, Department of Pathology, Anhui Medical University, The First Affiliated Hospital of Anhui Medical University, Hefei, 230601 Anhui China; 3 Department of Hematology, Tongling People’s Hospital, Tongling, 244000 Anhui China; 4 Department of Hematology, Jining NO. 1 People’s Hospital, Jining, 272000 Shandong China; 5 Department of Laboratory, Fuyang People’s Hospital, Fuyang, 236000 Anhui China; 6 ZENO Biotechnology (Shenzhen) Co, Shenzhen, 518000 Guangdong China; 7 Zeno Therapeutics Pte. Ltd., 600 North Bridge Road, Singapore, 188778 Singapore; 8 Eximmium Pte. Ltd., 600 North Bridge Road, Singapore, 188778 Singapore

**Keywords:** CAR-T target research, Gp350, EBV, Burkitt lymphoma, Immunotherapy

## Abstract

**Background:**

Epstein-Barr virus (EBV) is an oncovirus belonging to the herpesvirus family, associated with the pathogenesis of multiple malignancies, particularly Burkitt lymphoma (BL). The virus remains latent in host cells and plays a critical role in tumor progression through various mechanisms. A key glycoprotein, gp350, expressed during the lytic phase of EBV, is instrumental in viral entry into B cells and presents a unique antigenic target, making it a promising candidate for immunotherapeutic approaches, such as chimeric antigen receptor T-cell (CAR-T) therapy.

**Methods:**

In this study, we engineered CAR-T cells targeted against the gp350 glycoprotein and assessed their therapeutic potential through a series of in vitro and in vivo experiments. The efficacy of the gp350-CAR-T cells was evaluated by comparing their cytotoxic effects against both EBV-positive and -negative tumor cell lines. We utilized a xenograft model of Burkitt lymphoma to monitor the impact of gp350-CAR-T cell administration on tumor progression and overall survival.

**Results:**

The engineered gp350-CAR-T cells demonstrated potent cytotoxicity specifically against EBV-positive tumor cell lines. In our in vivo xenograft model, administration of gp350-CAR-T cells resulted in significant inhibition of tumor growth, highlighting their capability to effectively target and eliminate EBV-positive lymphomas. This selectivity underscores the potential of utilizing gp350 as a specific target for immunotherapy.

**Conclusion:**

Our findings advocate for the clinical application of gp350-directed CAR-T therapy as a prospective treatment strategy for patients with relapsed or refractory EBV-positive tumors. Given the encouraging preclinical results, further research is warranted to optimize CAR-T cell production processes and extend the potential of this therapy to other EBV-associated malignancies, paving the way for improved outcomes in affected patient populations.

**Supplementary Information:**

The online version contains supplementary material available at 10.1186/s12967-025-06188-w.

## Introduction

Epstein-Barr virus (EBV), a member of the herpesvirus family, is one of the most prevalent human pathogens, infecting over 90% of the global population. After primary infection, EBV establishes latency in B lymphocytes and typically remains asymptomatic in most individuals. However, it is associated with several malignancies, including Burkitt lymphoma (BL), Hodgkin lymphoma, nasopharyngeal carcinoma, and specific subtypes of gastric cancers [1].

Burkitt lymphoma is a highly aggressive B-cell tumor strongly linked to EBV, especially in endemic regions such as sub-Saharan Africa, where nearly all BL cases are EBV-positive [2–4]. EBV’s role in BL pathogenesis is closely tied to its ability to transform B cells and evade immune detection, resulting in unchecked proliferation of infected cells. Despite advancements in treating EBV-positive Burkitt lymphoma—such as intensive chemotherapy and monoclonal antibodies like rituximab—treatment remains challenging, particularly for patients with relapsed or refractory disease [3, 5]. While chemotherapy can be effective, it often leads to significant toxicity and the development of resistance. Moreover, EBV’s capacity for latency and immune evasion presents a major barrier to long-term disease control [1, 3]. These challenges have prompted the exploration of alternative therapeutic strategies, particularly those targeting the virus or viral proteins expressed on infected cells.

In recent years, immunotherapy—especially chimeric antigen receptor T-cell (CAR-T) therapy—has emerged as a promising strategy for treating hematological malignancies [6]. CAR-T therapy involves engineering a patient’s T cells to express synthetic receptors that bind to specific antigens on cancer cells, directing the immune system to attack and eliminate them. CD19-targeted CAR-T therapy has shown remarkable efficacy in certain B-cell malignancies, resulting in durable remissions for many patients with refractory disease [6, 7]. However, there is an urgent need for CAR-T therapies specifically targeting EBV-associated malignancies [8].

Gp350, an envelope glycoprotein expressed by EBV during its lytic phase [9–11], represents a key therapeutic target. Gp350 is essential for the interaction between EBV and the CD21 receptor on B cells, facilitating viral entry [12]. Its selective expression on EBV-infected cells makes it an ideal target for immunotherapy [13], enabling the development of therapies that specifically target EBV-positive cancer cells while sparing uninfected normal cells. Preclinical studies have demonstrated that gp350-targeted CAR-T cells exhibit potent cytotoxicity against EBV-positive lymphoblastoid cells and show efficacy in mouse models of EBV-associated malignancies [13, 14].

Despite these promising initial studies, the application of gp350-CAR-T therapy in EBV-positive Burkitt lymphoma remains largely underexplored. Most current research focuses on broader categories of EBV-associated lymphoproliferative disorders [15], with limited attention given to the specific challenges and therapeutic potential of Burkitt lymphoma. A significant gap in the literature is the lack of detailed in vivo studies assessing the efficacy and safety of gp350-CAR-T cells for EBV-positive Burkitt lymphoma, particularly in humanized mouse models or clinical trials. Furthermore, while in vitro studies have shown the cytotoxic potential of gp350-CAR-T cells, additional research is needed to validate these findings within more complex tumor microenvironments and to evaluate the specificity and long-term safety of this therapy.

This study aims to address these gaps by evaluating the therapeutic potential of gp350-targeted CAR-T cells for treating EBV-positive Burkitt lymphoma. The significance of this research lies in its potential to establish the efficacy and safety of gp350-targeted CAR-T therapy as an innovative treatment strategy for this malignancy. By specifically targeting a viral antigen, this approach could provide a more precise and less toxic alternative to conventional therapies, particularly for patients with relapsed or refractory disease. Additionally, this study could contribute to the broader development of viral antigen-targeted immunotherapies, opening new therapeutic avenues for various EBV-associated malignancies.

## Materials and methods

### Cell lines

Namalwa and RAJI are human-derived Burkitt lymphoma cell lines, while SUDHL-4 is a human-derived diffuse large B-cell lymphoma (DLBCL) cell line. The Namalwa and RAJI cell lines were obtained from Guangzhou Saiqu Biotechnology Co., Ltd., and the SUDHL-4 cell line was generously provided by Dr. Li from The First Hospital of Anhui Medical University, China. Both Namalwa and RAJI cell lines are EBV-positive, whereas the SUDHL-4 cell line is EBV-negative.

All cell lines were cultured in RPMI 1640 medium (Hyclone, Logan, UT, USA), supplemented with 10% heat-inactivated fetal bovine serum (FBS; EVERY GREEN, Zhejiang Tianhang Biotechnology Co., Ltd., China) and 1% antibiotics (100 U/mL penicillin G and 100 mg/mL streptomycin). The cells were maintained in a controlled environment using a cell incubator (Thermo Fisher Scientific Inc., USA) with 5% CO2 at 37 °C. Prior to experimentation, all cell lines underwent authentication through immunophenotyping to ensure their identity and integrity.

### Immunohistochemical staining

Tissue sections from patients with EBER-positive T-cell lymphoma, NK/T-cell lymphoma, and Burkitt lymphoma were utilized for gp350 immunohistochemistry (IHC) analysis, while sections from EBER-negative T-cell lymphoma and Burkitt lymphoma patients served as controls. All tissue samples were obtained from the Second Affiliated Hospital of Anhui Medical University, with ethical approval granted by its Medical Research Ethics Committee (Ethics Approval Number: YJ-YX2019-015). The gp350 antibody used for immunohistochemical staining was generously provided by Eximmium Pte. Ltd., Singapore.

For the IHC procedure, paraformaldehyde-fixed, paraffin-embedded tissue sections were baked at 60 °C for 60 min, dewaxed in xylene, and rehydrated through a graded ethanol series. Antigen retrieval was performed using a sodium citrate solution (pH 6) with a high-pressure method. Following this, endogenous peroxidase activity was blocked using 3% hydrogen peroxide, and non-specific binding was minimized with goat serum. Sections were then incubated overnight at 4 °C with a 1:50 dilution of a monoclonal antibody against human gp350.

After returning to room temperature, goat anti-rat IgG polymer (secondary antibody; Bioss, China) was added and incubated at 37 °C for 30 min. Color development was achieved using DAB (immunostaining kit; CELNOVTE Henan Celnovte Biotechnology Co., Ltd., China), followed by hematoxylin counterstaining. Typical fields at 40 × magnification was selected under a microscope and independently analyzed by two pathologists for confirmation of results.

### Flow cytometry analysis

Three cell lines—Namalwa, RAJI, and SUDHL-4—were collected during the logarithmic growth phase in 500 μL of culture medium, followed by the addition of 1 mL of PBS. The cells were centrifuged at 1500 rpm for 5 min, and the supernatant was discarded. For each specimen, two tubes were prepared, adjusting the nucleated cell count to 5–8 × 10^5 per tube. Each tube received the gp350 antibody and an isotype control (ZENO, Singapore), followed by a 30 min incubation at room temperature.

After incubation, 1 mL of PBS was added to each tube, and the cells were washed three times before being centrifuged again at 1500 rpm for 5 min. The cells were then resuspended in 100 μL of PBS. Next, donkey anti-rat IgG antibody conjugated to Alexa Fluor 488 (Abcam, Cambridge, UK) and CD19-PC7 fluorescent-labeled monoclonal antibody (Beckman Coulter, Miami, FL, USA) were added and incubated for an additional 30 min. Following this incubation, 1 mL of PBS was added, and the cells were centrifuged once more at 1500 rpm for 5 min to remove excess antibodies.

For flow cytometry analysis, the supernatant was discarded, and the cells were resuspended in an appropriate volume of PBS. Flow cytometry was performed using a three-color fluorescent labeling protocol (FITC, APC, and PC7) to analyze gp350 expression. Data acquisition and analysis were conducted using a Cytoflex S flow cytometer (Beckman Coulter) and CytExpert software (Beckman Coulter).

### Confocal microscopy detection

Cells from three cell lines—Namalwa, RAJI, and SUDHL-4—were collected during the logarithmic growth phase in 1 mL of culture medium. The cells were centrifuged at 1000 rpm for 5 min, and the supernatant was discarded. The cell pellet was resuspended in 3 mL of PBS, washed once, and centrifuged again at 1000 rpm for 5 min. The resulting pellet was resuspended in 300 μL of PBS and evenly smeared onto glass slides, which were then fixed with formaldehyde for 30 min. Following fixation, the slides were rinsed four times with PBST, with each rinse lasting 5 min. To block non-specific binding, goat serum (Zhongshan Golden Bridge, China) was applied at room temperature for 60 min. After removing the blocking buffer, the gp350 primary antibody was added, and the slides were incubated overnight at 4 °C.

After incubation, the slides were allowed to return to room temperature and were washed four times with PBST, with each wash lasting 5 min. A fluorescently labeled secondary antibody (donkey anti-rat IgG antibody conjugated to Alexa Fluor 488) was then applied, and the slides were incubated in the dark at room temperature for 3 h. The slides were rinsed four more times with PBST, ensuring that they remained in the dark throughout the process. Finally, excess liquid was removed from the slides, and 50 μL of anti-fade mounting medium containing DAPI (Beyotime, China) was applied. The slides were then examined using a confocal microscope (Zeiss, Germany).

### Western blot analysis

Cells from three cell lines were collected, and the resulting pellets were immediately placed on ice. The cell pellets were washed twice with pre-chilled PBS buffer. For each sample, 80–120 μL of lysis buffer (RIPA: PMSF = 100:1) was added, and the cells were lysed on ice for 50 min. The lysates were then centrifuged at 12,000 rpm for 20 min at 4 °C, and the supernatant was carefully transferred to a fresh Eppendorf tube.

Protein concentrations were measured using a BCA protein assay kit (Beyotime, China) according to the manufacturer’s instructions. Loading buffer (Beyotime, China) was added to each sample, and the proteins were denatured by heating at 100 °C for 10 min. A total of 20 μg of protein per sample was loaded onto an SDS-PAGE gel, and electrophoresis was performed at a constant voltage of 170 V for 60 min.

Following electrophoresis, proteins were transferred onto a PVDF membrane at a constant current of 200 mA for 60 min. The membrane was then blocked in TBST containing 5% skim milk on a shaker at room temperature for 2 h. After blocking, the membrane was washed three times with TBST, with each wash lasting 10 min. The membrane was incubated overnight at 4 °C with the gp350 antibody diluted to 1:1000.

After incubation, the membrane was washed three additional times with TBST, each wash lasting 10 min. A goat anti-rat IgG secondary antibody diluted to 1:2000 was then added, and the membrane was incubated on a shaker at room temperature for 1 h.

### Preparation of gp350-CAR-T cells

Peripheral blood T lymphocytes were isolated from healthy adults using density gradient centrifugation and subsequently resuspended in PRIME-XV medium (Fujifilm, USA) at a concentration of 2.0 × 10^6 cells/mL. Anti-CD3 and anti-CD28 antibodies (Miltenyi, Germany) were added to achieve a final concentration of 100 ng/mL, along with IL-7 and IL-15 (AcroBiosystems, China) at 10 μg/mL. One milliliter of the suspension was reserved as a control for Mock T cells in flow cytometry, while the remaining cells were cultured at 37 °C in a 5% CO2 atmosphere. After 24 h, the gp350 CAR lentiviral vector (Genscript, China) was introduced at a multiplicity of infection (MOI) of 5, supplemented with human serum albumin (Baxalta, USA) at a final concentration of 2.5%. The structure of the anti-gp350 CAR is depicted in Figure S1, where the gp350 monoclonal antibody is expressed as a single-chain variable fragment (scFv). Following another 24 h incubation period with the viral vector, the medium was replaced after centrifugation, and the cells were resuspended in fresh PRIME-XV medium containing IL-7 and IL-15 to maintain a concentration of 0.8 × 10^6 cells/mL. Cells were sampled every 2–3 days for counting and medium replenishment. On day 4, flow cytometry was conducted to detect CAR + T cells using Alexa Fluor^®^ 647 AffiniPure F(ab’)₂ Fragment Donkey Anti-Rat IgG (Jackson ImmunoResearch, USA), as well as to assess T helper (Th) and cytotoxic T lymphocyte (CTL) populations using CD3, CD4, CD8, and CD45 antibodies (Invitrogen, USA). The gating strategy is as follows: 1.Lymphocyte selection (Figure S2A,F): Lymphocyte populations were gated in the FSC vs. SSC scatter plot to exclude debris and non-lymphocyte populations. 2. Dead cell exclusion (Figure S2B,G): Based on the selected lymphocyte populations, dead cells were excluded using the FVD-KO viability dye, and live cell populations were obtained. 3.T-cell identification (Figure S2C,H): Within the live cell population, T cells were gated using CD3 antibody staining. 4.CAR expression detection (Figure S2D, I): In the CD3⁺ T-cell population, CAR expression was assessed using an antibody recognizing the CAR-specific Fab fragment (APC fluorescence), with the MOCK-T group serving as the negative control. 5.CD4/CD8 subset analysis (Figure S2E, J): CD4⁺ and CD8⁺ subsets were distinguished within the T-cell population using CD4-FITC and CD8-PE double staining. This gating strategy enables precise identification of target cell populations, providing a reliable foundation for subsequent experiments.Cells were harvested between days 6 and 8 for final flow cytometry analysis to evaluate CAR-T expression and the percentages of Th and CTL populations.

### Flow cytometry assessment of CAR-T cell cytotoxicity on tumor cells

Effector and target cells were prepared at a density of 1.0 × 10^6 cells/mL. MOCK-T or gp350-CAR-T cells were co-cultured with Namalwa, RAJI, and SUDHL-4 tumor cells at effector-to-target (E:T) ratios of 1:1 and 2:1. The appropriate numbers of effector and target cells were added to 48-well plates, with three replicates per group. Culture medium was then added to each well to achieve a final volume of 500 μL, followed by gentle mixing of the plates. The plates were incubated at 37 °C for both 0 and 24 h. At each time point, cells were collected and resuspended to create a single-cell suspension, followed by centrifugation at 300 g for 5 min. The supernatants were collected, and the cell pellets were resuspended in 100 μL of PBS for cell counting. To analyze the populations, APC-CD19, FITC-CD3, and PC7-CD45 antibodies were added to the resuspended cells, which were then incubated in the dark at room temperature for 15 min. After incubation, the cells were washed with PBS, centrifuged again, and resuspended in 200 μL of PBS. Flow cytometry was performed to evaluate cytotoxicity based on the expression of surface markers in the effector and target cell populations.

### Analysis of secreted IFN-γ and TNF-α using ELISA

Gp350-CAR-T and MOCK-T cells were co-cultured with tumor cells at various effector-to-target (E:T) ratios for 24 h. Following this incubation, supernatants were collected, and levels of IFN-γ and TNF-α were measured using ELISA kits (Linc-Bio, Hangzhou, China) according to the manufacturer’s protocol. Cytokine concentrations were quantified using an Absorbance Reader (BioTek, Vermont, USA).

### In vivo proliferation assay

Three-week-old BALB/C nude mice (16–23 g) were obtained from Beijing Viton Lihua Laboratory Animal Technology Co. and housed in a Specific Pathogen-Free (SPF) facility at the Institute of Advanced Technology, University of Science and Technology of China. After a one-week acclimation period, 1.0 × 10^7 Namalwa cells were resuspended in 200 μL of PBS and injected subcutaneously into the right axilla to establish a Burkitt lymphoma xenograft model. We chose Namalwa over the Raji cell line due to its higher in vivo tumor formation rate and more pronounced tumor growth potential.

The mice were randomly divided into two groups: the gp350-CAR-T group (n = 4) and the MOCK-T group (n = 4). On day 4, each mouse received an intravenous injection of 5 × 10^6 gp350-CAR-T or MOCK-T cells via the tail vein. Body weight and tumor dimensions (length and width) were recorded every other day, with tumor volume calculated using the formula (length × width × width)/2. When tumor volume reached approximately 1500 mm^3^, the mice were euthanized, and tumors were excised. Changes in tumor volume and weight were recorded and plotted over time.

Tumor specimens were collected for hematoxylin and eosin (HE) staining to assess morphology, and immunohistochemistry was performed to evaluate the expression of Ki-67, BCL-2, Bax, gp350, and CD3. Typical fields at 40 × magnification was selected under a microscope and independently analyzed by two pathologists for confirmation. The staining intensity of the immunohistochemical markers was quantified using Fiji (ImageJ) software with the IHC Toolbox plugin.

### In vivo migration assays and bioluminescent imaging

Four-week-old female NOD-SCID mice (14–16 g) were obtained from Beijing Viton Lihua Laboratory Animal Technology Co. Namalwa cells (5.0 × 10^6) or SUDHL-4 cells (1.5 × 10^7), labeled with fluorescein, were resuspended in 200 μL of PBS and injected via the tail vein. The mice were divided into two groups based on the cell line: Namalwa and SUDHL-4, with further subdivisions into three groups: the blank group (Namalwa, n = 3; SUDHL-4, n = 4), the MOCK-T group (Namalwa, n = 3; SUDHL-4, n = 4), and the gp350-CAR-T group (Namalwa, n = 3; SUDHL-4, n = 4).

On day 4, each mouse received an intravenous injection of either 1 × 10^7 MOCK-T or gp350-CAR-T cells via the tail vein, while the blank group received an equivalent volume of PBS. Bioluminescent images were captured using the IVIS Spectrum system (PerkinElmer) to monitor cell migration. Imaging was conducted under 2% isoflurane anesthesia, with images taken 5 min after intraperitoneal injection of 3 mg D-luciferin per mouse. Mice were monitored daily for changes in weight and clinical signs of metastasis, such as piloerection and significant weight loss. Euthanasia was performed in accordance with ethical guidelines upon observation of signs of metastasis.

### Statistical analyses

Statistical analyses were performed using GraphPad Prism 9.0. Continuous variables are presented as mean ± standard deviation (SD). The Shapiro–Wilk test was utilized to evaluate the normality of data distribution.

For comparisons between two groups, an unpaired t-test was applied to normally distributed data. When assessing multiple groups with normally distributed data, two-way ANOVA was employed, followed by Sidak’s multiple comparisons test for post-hoc analysis.

Survival data were analyzed using Kaplan–Meier curves, with group comparisons conducted via the log-rank test. Statistical significance was defined as follows: *p < 0.05; **p < 0.01; ***p < 0.001; and ****p < 0.0001.

## Results

### gp350 expression in lymphoma patients

To evaluate gp350 protein expression levels in EBER + and EBER- lymphoma patients, we conducted immunohistochemical staining on various lymphoma tissue samples. The selected specimens included tissue sections from patients with EBER + T-cell lymphoma, NK/T-cell lymphoma, and Burkitt lymphoma. Control samples comprised tissue sections from EBER- patients with the same types of lymphoma.

**Fig. 1:**
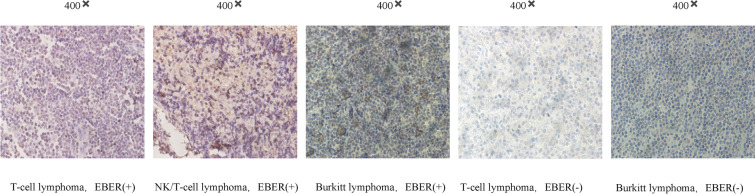
gp350 Expression in Lymphoma Tissue Samples. Immunohistochemical staining was conducted on tissue sections from patients with EBER-positive T-cell lymphoma, NK/T-cell lymphoma, and Burkitt lymphoma, along with EBER-negative controls for each lymphoma type. The results demonstrated gp350 expression in all EBER-positive samples, irrespective of the lymphoma subtype. In contrast, no gp350 expression was observed in the EBER-negative samples. These findings underscore the association between gp350 expression and EBV infection in lymphoma tissues. Representative images were captured at 400 × magnification

The immunohistochemical staining results revealed robust expression of gp350 protein in all EBER + lymphoma samples, irrespective of the lymphoma subtype. In stark contrast, gp350 expression was absent in the EBER- patient samples (Fig. 1). These findings indicate a strong correlation between gp350 expression and EBV infection in lymphoma tissues.

We further analyzed gp350 protein expression using flow cytometry in EBV + and EBV- cell lines. Three cell lines were evaluated: Namalwa and RAJI (EBV + Burkitt lymphoma cell lines) and SUDHL-4 (an EBV- diffuse large B-cell lymphoma cell line). Cells were stained with a gp350-specific antibody and compared to an isotype control.

**Fig. 2:**
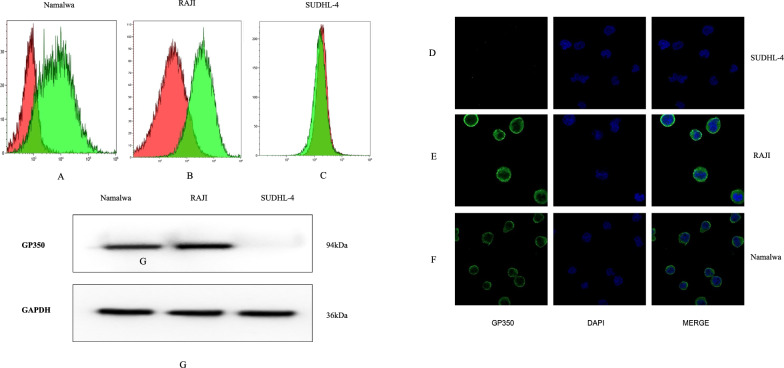
gp350 Expression in EBV + and EBV- Cell Lines. **A**, **B**, **C** Flow cytometry analysis was conducted to assess gp350 expression in three cell lines: Namalwa and RAJI (both EBV-positive Burkitt lymphoma cell lines) and SUDHL-4 (an EBV-negative diffuse large B-cell lymphoma cell line). The cells were stained with gp350-specific antibodies, labeled in green, and compared to isotype controls, labeled in red. A significant shift in fluorescence intensity was observed in Namalwa and RAJI cells, indicating the presence of gp350 expression. In contrast, SUDHL-4 cells exhibited no gp350 expression, as their fluorescence intensity overlapped with that of the isotype control. **D**, **E**, **F** Microscopy was employed to assess gp350 expression in the Namalwa and RAJI (EBV-positive) and SUDHL-4 (EBV-negative) cell lines. The cells were stained with gp350-specific antibodies, highlighted in green, and DAPI, marked in blue, to visualize the nuclei. In Namalwa and RAJI cells, gp350 expression was prominently localized to the cell membrane. In contrast, SUDHL-4 cells showed no detectable gp350 expression. **G** Immunoblotting was conducted to detect gp350 protein in the Namalwa and RAJI (EBV-positive) and SUDHL-4 (EBV-negative) cell lines. A prominent gp350 band, approximately 94 kDa, was observed in the EBV-positive cell lines, Namalwa and RAJI. In contrast, no gp350 expression was detected in the SUDHL-4 cell line. GAPDH, at approximately 36 kDa, was used as a loading control to ensure equal protein loading across samples

In both Namalwa and RAJI cells, the gp350-positive population exhibited a significant shift in fluorescence intensity compared to the isotype control, as evidenced by the clear separation between the green (gp350-antibody labeled) and red (isotype control) peaks (Fig. 2A, B). This distinct difference in fluorescence intensity confirms high levels of gp350 protein expression in EBV-positive cell lines. Conversely, the SUDHL-4 cell line displayed minimal gp350 expression, with fluorescence intensity largely overlapping with that of the isotype control (Fig. 2C), indicating undetectable levels of gp350 protein.

Confocal microscopy was employed to analyze both the expression and localization of gp350 protein in EBV + and EBV- cell lines. Namalwa and RAJI (EBV + Burkitt lymphoma cell lines) were stained with a gp350-specific antibody (green) and counterstained with DAPI (blue) to visualize nuclei. In Namalwa and RAJI cells, strong green fluorescence was observed, primarily localized to the cell membrane, confirming gp350 protein expression in EBV + cells (Fig. 2E, F). DAPI staining validated proper nuclear visualization and demonstrated normal cell morphology. Merged images indicated colocalization of gp350 with the cellular membrane in these EBV + cell lines. In contrast, SUDHL-4 cells, which are EBV-, exhibited no detectable green fluorescence, indicating an absence of gp350 protein expression (Fig. 2D). DAPI staining confirmed intact nuclei in SUDHL-4 cells, similar to those in EBV + cell lines, but without any detection of gp350.

Western blot analysis was conducted to further evaluate gp350 protein expression across EBV + and EBV- cell lines. The same three cell lines—Namalwa and RAJI (EBV-positive Burkitt lymphoma) and SUDHL-4 (EBV-negative diffuse large B-cell lymphoma)—were assessed using gp350-specific antibodies, with GAPDH serving as a loading control.

The results demonstrated a strong band at approximately 94 kDa in both Namalwa and RAJI cell lines, indicating high levels of gp350 protein expression in these EBV + cells. In contrast, the SUDHL-4 cell line showed no detectable gp350 expression, as evidenced by the absence of a band at the corresponding molecular weight (Fig. 2G). GAPDH was consistently detected at approximately 36 kDa across all samples, confirming equal protein loading. These collective results validate that gp350 protein is specifically expressed in EBV + Burkitt lymphoma cell lines while being absent in EBV- cells. The strong correlation between gp350 expression and EBV infection underscores its potential as a therapeutic target for treating EBV-associated malignancies.

### Transduction efficiency of gp350-targeted CAR-T cells

This study employed flow cytometry (FCM) to assess the viability and transduction efficiency of CAR-T cells following their expansion. The viability of CAR-T and MOCK-T cells post-expansion was evaluated using FVD staining, while donkey anti-rat IgG was utilized to detect CAR-T transduction rates. The flow cytometry results revealed that the viability of CAR-T and MOCK-T cells after transduction was 92.73% and 92.89%, respectively. Additionally, CAR-T cells exhibited a transduction rate of 41.68% (which refers specifically to the batch of CAR-T cells used in this experiment), with CD4 + T cells showing a transduction rate of 33.02% and CD8 + T cells demonstrating a significantly higher rate of 60.61% (Figure S2). These findings indicate successful expansion and transduction of CAR-T cells, highlighting the potential for effective therapeutic application.

### Cytotoxicity of gp350-CAR-T cells against tumor cells

**Fig. 3:**
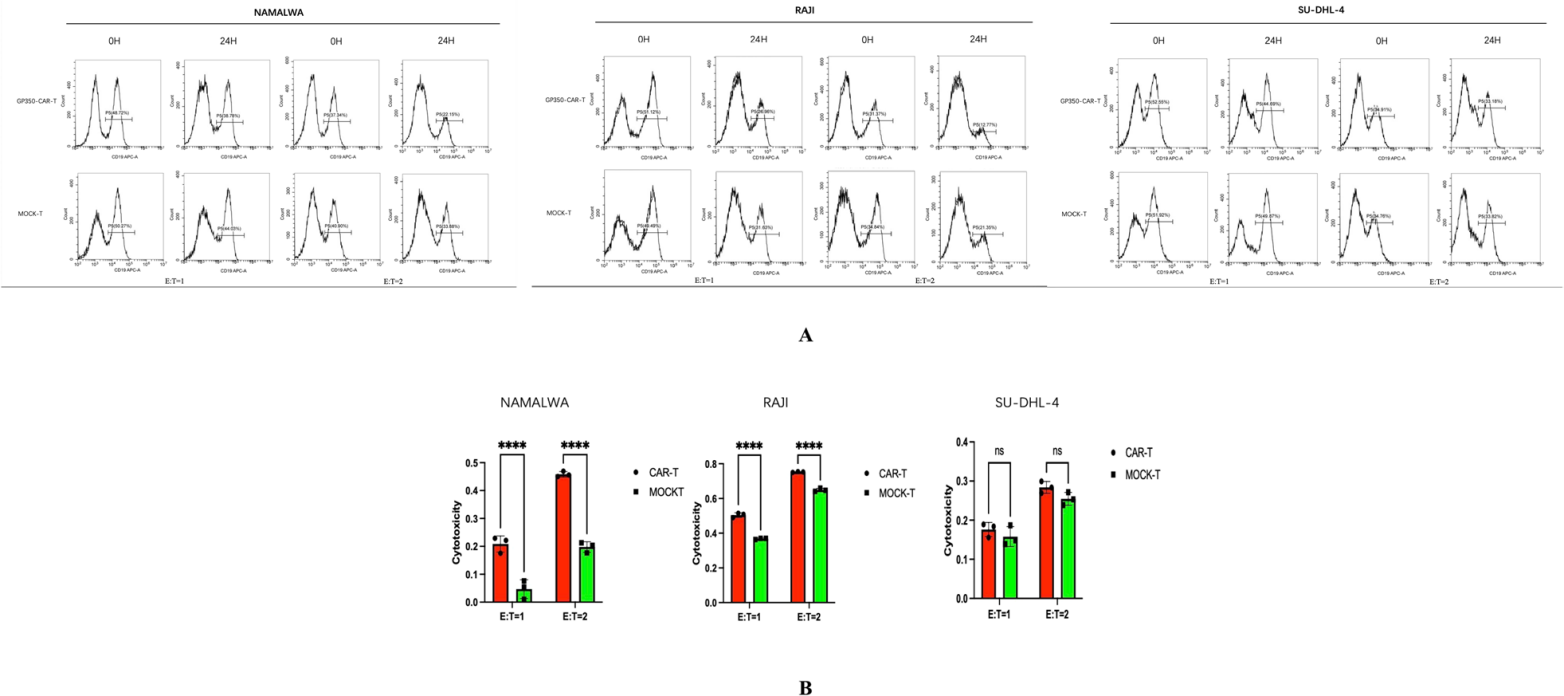
Cytotoxicity of gp350-CAR-T Cells in Co-culture. **A** gp350-CAR-T cells and Mock-T cells were co-cultured with Namalwa and RAJI (EBV-positive) cell lines, as well as the SUDHL-4 (EBV-negative) cell line, at effector-to-target (E:T) ratios of 1:1 and 2:1. The gp350-CAR-T cells demonstrated significant cytotoxicity against the Namalwa and RAJI cells, markedly reducing the tumor cell populations within 24 h. In contrast, minimal cytotoxicity was observed in the SUDHL-4 cell line, indicating the specificity of gp350-CAR-T cells for EBV-positive targets. **B** The bar chart illustrates cytotoxicity across various cell lines at the 24-h mark. It quantifies the results, showing that gp350-CAR-T cells exhibited significantly higher killing rates in EBV-positive cell lines compared to Mock-T cells (P < 0.05). In contrast, no significant difference in cytotoxicity was observed in the SUDHL-4 cell line, highlighting the specificity of gp350-CAR-T cells for EBV-positive targets

Flow cytometry was employed to evaluate the cytotoxicity of gp350-CAR-T cells against both EBV + and EBV- tumor cell lines, specifically Namalwa, RAJI, and SUDHL-4. gp350-CAR-T cells and MOCK-T cells were co-cultured with tumor cells at different effector-to-target (E:T) ratios of 1:1 and 2:1 for a duration of 24 h. The results demonstrated significant cytotoxicity of gp350-CAR-T cells against EBV + Namalwa and RAJI cells, while showing minimal cytotoxicity against the EBV- SUDHL-4 cell line. After 24 h of co-culture, gp350-CAR-T cells markedly reduced the number of CD19 + tumor cells in both Namalwa and RAJI lines (Fig. 3A). At the 2:1 E:T ratio, the reduction in the CD19 + population was particularly pronounced, as indicated by substantial decreases in flow cytometry plots. Although the cytotoxicity at the 1:1 E:T ratio remained significant, it was less pronounced compared to the higher E:T ratio, suggesting a possible dose-dependent effect of gp350-CAR-T cells. In contrast, the SUDHL-4 (EBV-) cell line exhibited minimal tumor cell lysis when co-cultured with gp350-CAR-T cells. Flow cytometry analysis indicated that the CD19 + population in SUDHL-4 cells remained largely unchanged even at the 2:1 E:T ratio (Fig. 3A). This finding highlights the specificity of gp350-CAR-T cells in targeting EBV + tumor cells. Statistical analysis confirmed that the cytotoxicity of gp350-CAR-T cells was significantly greater than that of MOCK-T cells, with a p-value of less than 0.05 for both Namalwa and RAJI cell lines (Fig. 3B). These results underscore the effectiveness of gp350-CAR-T cells in selectively targeting and eliminating EBV + Burkitt lymphoma cells.

### Cytokine secretion analysis of gp350-CAR-T cells

**Fig. 4:**
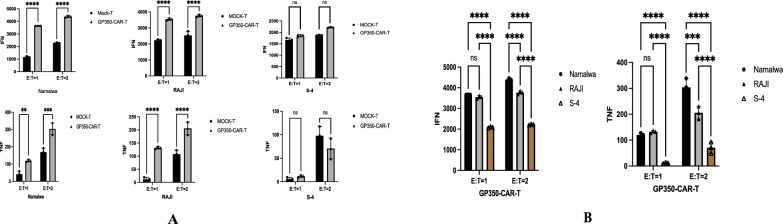
Cytokine Secretion of gp350-CAR-T Cells in Co-culture. **A** gp350-CAR-T cells and Mock-T cells were co-cultured with Namalwa and RAJI (EBV-positive) cell lines, as well as the SUDHL-4 (EBV-negative) cell line, at effector-to-target (E:T) ratios of 1:1 and 2:1. The gp350-CAR-T cells exhibited significantly higher cytokine secretion, specifically IFN-γ and TNF-α (measured in pg/ml), when interacting with the EBV-positive cell lines. In contrast, minimal cytokine release was observed in the EBV-negative SUDHL-4 cell line, highlighting the targeted response of gp350-CAR-T cells to EBV-positive targets. **B** The bar chart illustrates cytokine secretion by gp350-CAR-T cells across various tumor cell lines. These cells demonstrated significantly enhanced cytotoxicity and cytokine secretion, including IFN-γ and TNF-α (measured in pg/ml), especially at different effector-to-target (E:T) ratios in EBV-positive cell lines. The differences in cytokine levels were statistically significant (P < 0.05). **A**, **B** BALB/C nude female mice were subcutaneously injected with Namalwa cells to establish Burkitt lymphoma xenografts. The mice were then treated with either gp350-CAR-T cells or Mock-T cells via tail vein injection. In the gp350-CAR-T-treated group, both tumor volume and weight were significantly reduced compared to the Mock-T group, with these differences being statistically significant (P < 0.05)

The cytokine release from gp350-CAR-T and MOCK-T cells was analyzed after 24 h of co-culture with Namalwa, RAJI, and SUDHL-4 tumor cell lines at different effector-to-target (E:T) ratios of 1:1 and 2:1. The levels of IFN-γ and TNF-α in the supernatants were quantified using ELISA, and statistical analysis was performed to assess the significance of the results (P < 0.05) (Fig. 4A). The results demonstrated that gp350-CAR-T cells secreted significantly higher levels of IFN-γ and TNF-α compared to MOCK-T cells when co-cultured with EBV + Namalwa and RAJI cells. At the 2:1 E:T ratio, cytokine levels were markedly elevated, indicating strong activation and an enhanced immune response of gp350-CAR-T cells against EBV + tumor cells. At the 1:1 E:T ratio, while cytokine secretion remained elevated, it was lower than that observed at the 2:1 ratio, demonstrating a dose-dependent release of cytokines. In contrast, when co-cultured with the EBV- SUDHL-4 cell line, gp350-CAR-T cells did not exhibit significant cytokine release. The levels of IFN-γ and TNF-α remained low and comparable to those of MOCK-T cells, as shown in the ELISA results. Notably, gp350-CAR-T cells secreted significantly higher levels of IFN-γ and TNF-α in EBV-positive cell lines (Namalwa and RAJI) compared to the EBV-negative SUDHL-4 (S-4). The secretion was more pronounced at the 2:1 E:T ratio than at the 1:1 ratio (Fig. 4B).These findings further confirm the efficacy of gp350-CAR-T cells in recognizing and responding to EBV + Burkitt lymphoma cells, as evidenced by their robust cytokine release.

### Gp350-CAR-T cells inhibits subcutaneous tumor growth

**Fig. 5:**
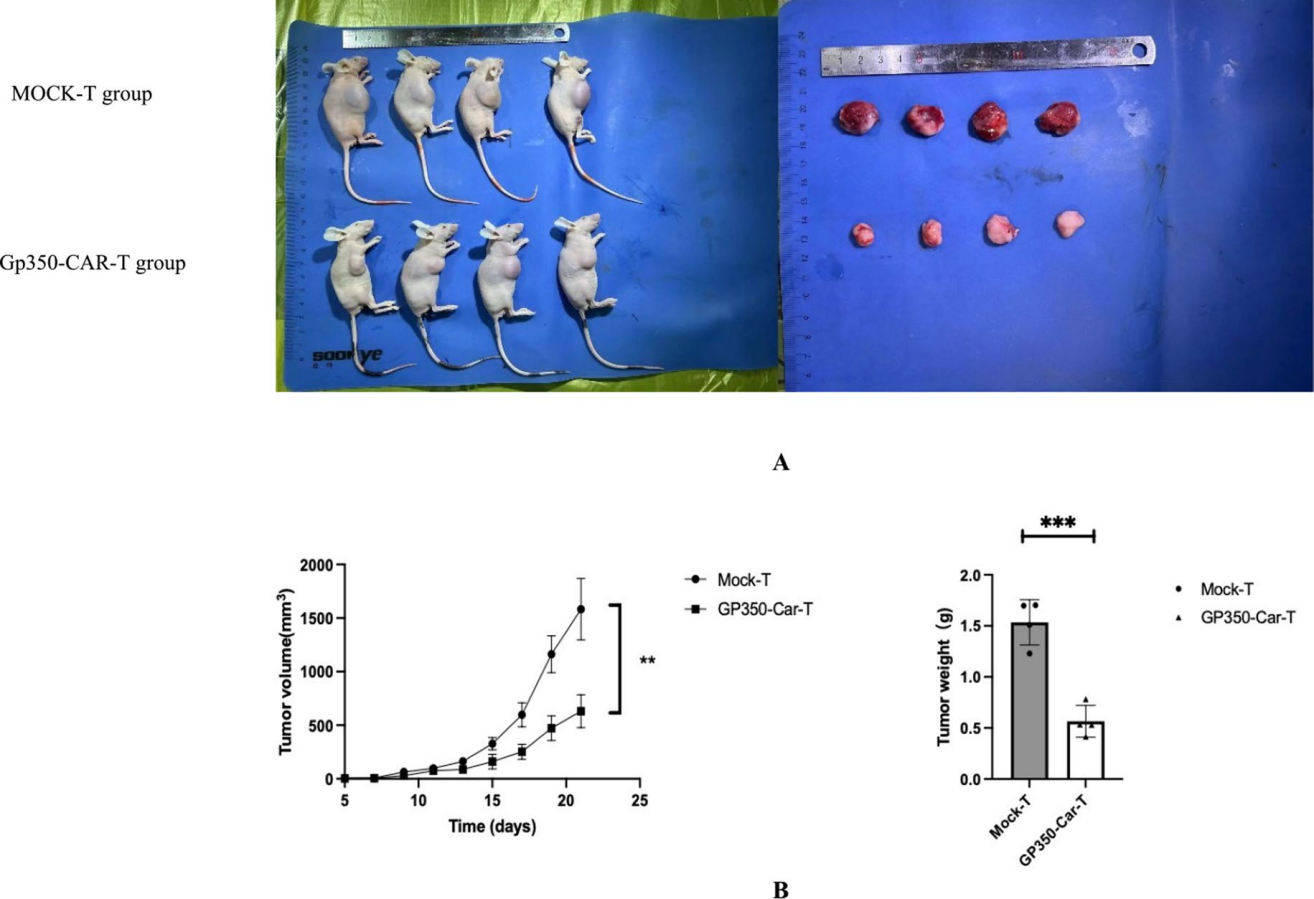
Subcutaneous Tumor Growth Experiment in BALB/C Nude Mice. **A**, **B** BALB/C nude female mice were subcutaneously injected with Namalwa cells to establish Burkitt lymphoma xenografts. Mice were treated with GP350-CAR-T or Mock-T cells via tail vein injection. Tumor volume and weight were significantly reduced in the GP350-CAR-T-treated group compared to the Mock-T group, with the differences showing statistical significance (P < 0.05)

To evaluate the anti-tumor activity of gp350-CAR-T cells, we inoculated 1.0 × 10^7 Namalwa cells subcutaneously into the right axilla of nude mice. On day 4, 5 × 10^6 gp350-CAR-T or MOCK-T cells were injected via the tail vein. Tumor volume was measured every other day. The results demonstrated a significant reduction in tumor volume and weight in the gp350-CAR-T group compared to the MOCK-T group (Fig. 5A, B). When the tumor volume reached approximately 1500 mm^3^, the mice were euthanized, and tumors were excised and weighed.

Histological analysis of hematoxylin and eosin (HE)-stained tumor tissues revealed pronounced differences between the groups. The MOCK-T group exhibited significant tumor cell proliferation, characterized by tightly packed cells with irregular morphology and a high nucleus-to-cytoplasm ratio. In contrast, the gp350-CAR-T group displayed extensive tumor cell necrosis, with loosely arranged cells and areas of apoptosis, indicating a potent inhibitory effect of gp350-CAR-T cells on tumor growth.

Immunohistochemical analysis further supported these findings, revealing that Ki-67 and BCL-2 levels were significantly higher in the MOCK-T group compared to the gp350-CAR-T group. This suggests greater proliferative activity and anti-apoptotic properties in MOCK-T tumors. Conversely, Bax expression was markedly elevated in the gp350-CAR-T group, indicating enhanced apoptosis. Additionally, the gp350-CAR-T group demonstrated a substantial increase in CD3-positive T cell infiltration, which predominantly consisted of CAR-positive cells as confirmed by IHC results, further corroborating the anti-tumor efficacy of gp350-CAR-T cells.

Notably, gp350 expression was significantly higher in the MOCK-T group than in the gp350-CAR-T group, suggesting that tumor reduction in the gp350-CAR-T group was primarily due to targeted killing of gp350-expressing cells (Figure S3). These findings validate the specific mechanism of action of gp350-CAR-T cells and underscore their potential as an effective therapeutic strategy against EBV-associated tumors.

### Gp350-CAR-T cells significantly inhibits tumor dissemination

**Fig. 6:**
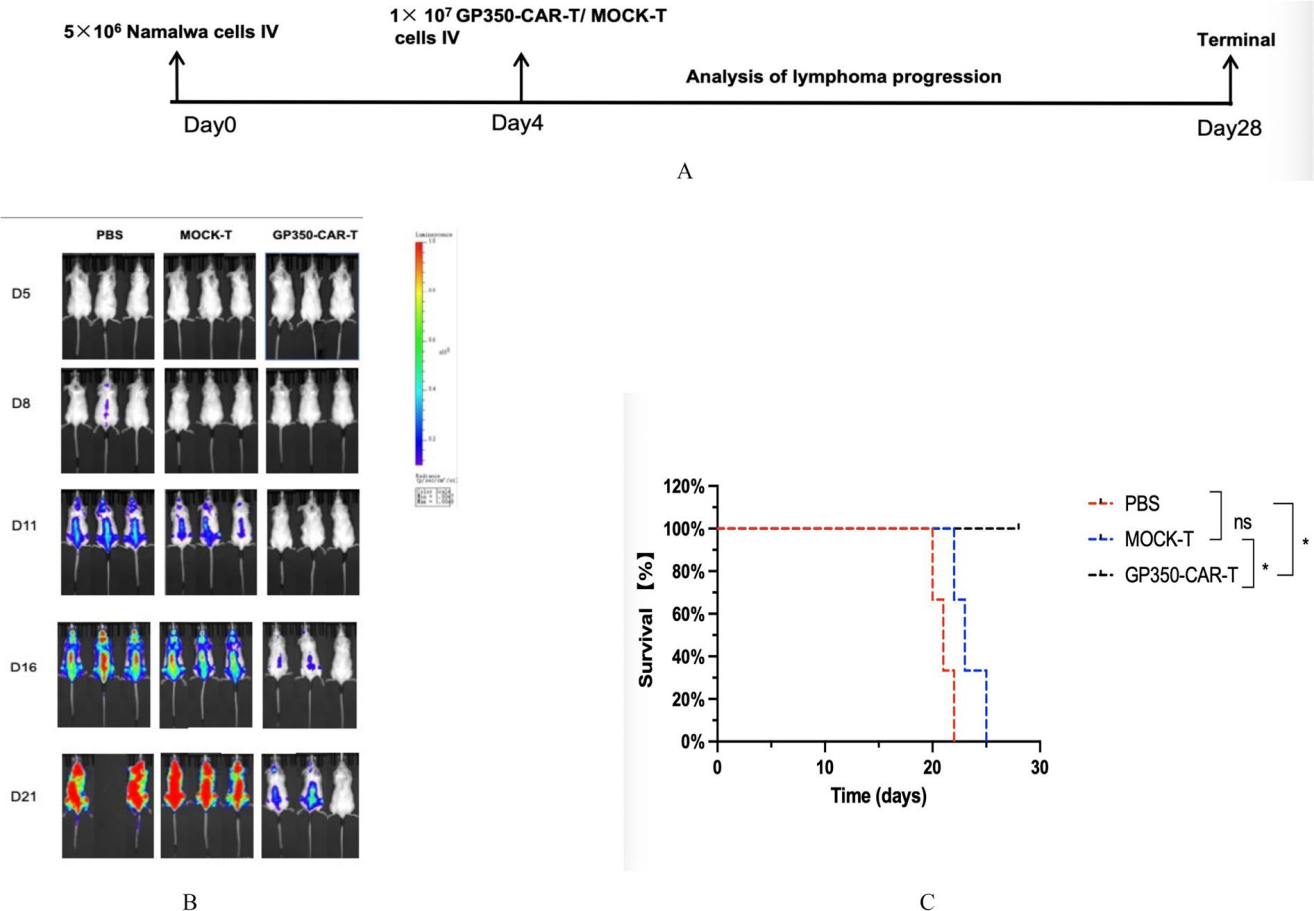
Tumor Dissemination Experiment of Namalwa Cells in NOD-SCID Mice. **A** Schematic Diagram of the Experiment **B** NOD-SCID mice were intravenously injected with gp350-positive Namalwa cells to establish a tumor dissemination model. The mice were then treated with gp350-CAR-T cells, Mock-T cells, or PBS. Bioluminescence imaging revealed that gp350-CAR-T cells significantly inhibited tumor dissemination compared to both the Mock-T and PBS groups. **C** The survival rate in the gp350-CAR-T-treated group was significantly higher, with clear statistical differences observed among the three groups (P < 0.05)

We have demonstrated that gp350-CAR-T cells can effectively inhibit tumor growth in vivo, prompting us to investigate whether these cells can also prevent tumor dissemination. A total of 5 × 10^6 fluorescently labeled Namalwa cells were resuspended in 200 μL of PBS and injected into NOD-SCID mice via the tail vein (Fig. 6A). The mice were randomly divided into three groups: a blank group (n = 3), a MOCK-T group (n = 3), and a gp350-CAR-T group (n = 3). On day 4, each mouse received an intravenous injection of either 1 × 10^7 MOCK-T or gp350-CAR-T cells, while the blank group received an equivalent volume of PBS.

Bioluminescence imaging was performed to monitor tumor dissemination and Living Image software was used to quantify fluorescence intensity in metastatic organs. By day 11, differences in tumor dissemination began to emerge, with extensive spread observed in both the blank and MOCK-T groups, particularly affecting parenchymal organs (Fig. 6B). Over time, these differences became increasingly pronounced.

We also compared the survival curves of the three groups. Survival analysis revealed significant differences between the gp350-CAR-T group, the MOCK-T group, and the PBS control group (Fig. 6C). The gp350-CAR-T group exhibited significantly prolonged survival compared to both the MOCK-T and PBS groups. By day 28, all mice in the MOCK-T and PBS groups had succumbed to tumor burden, while mice in the gp350-CAR-T group remained alive. On day 28, the surviving mice in the gp350-CAR-T group were euthanized. These results confirm that gp350-CAR-T cells significantly inhibit the dissemination of EBV + tumor cells in vivo.

**Fig. 7:**
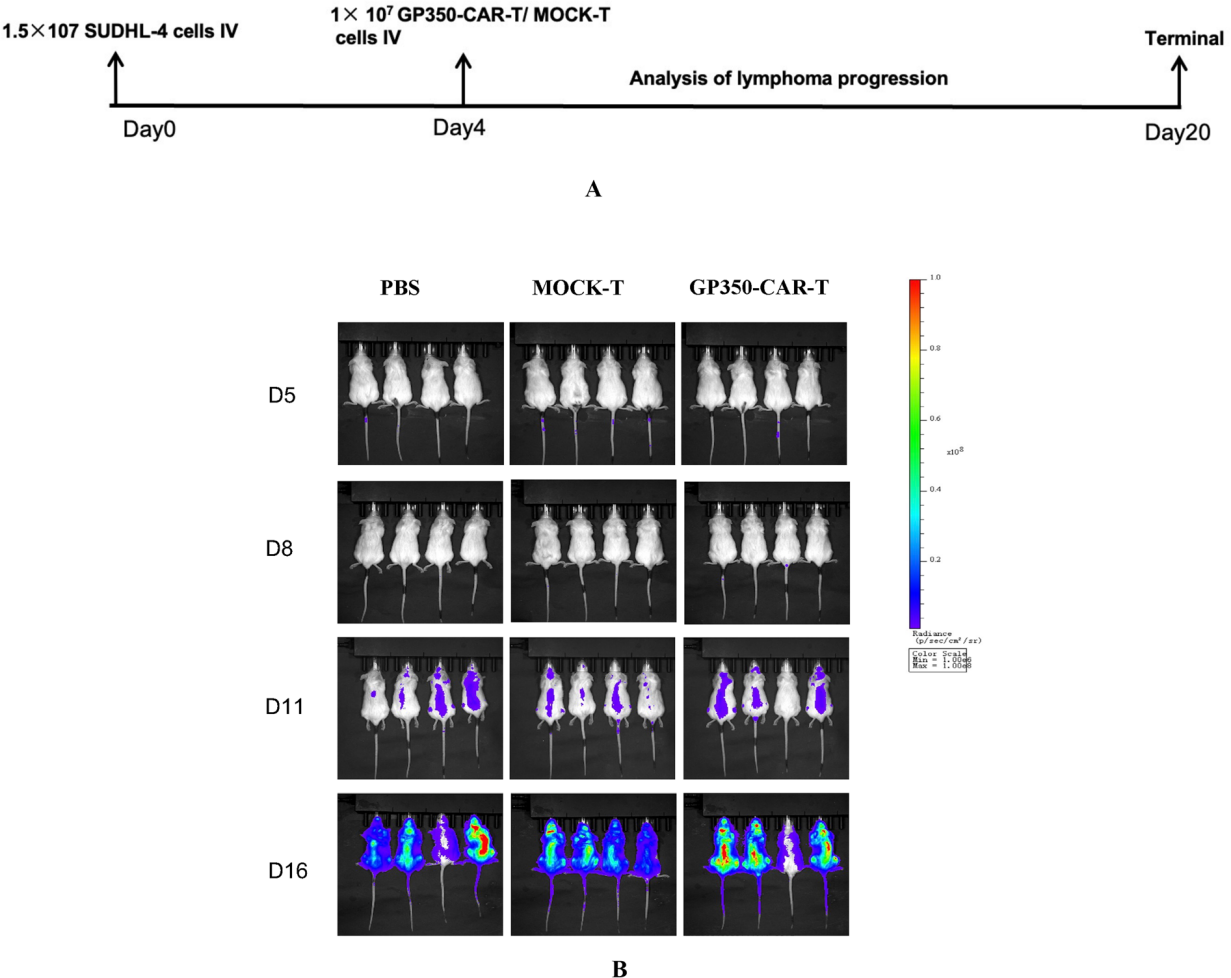
Tumor Dissemination Experiment of SUDHL-4 Cells in NOD-SCID Mice. **A** Schematic Diagram of the Experiment **B** NOD-SCID mice were intravenously injected with EBV-negative SUDHL-4 cells to establish a tumor dissemination model. The mice were then treated with gp350-CAR-T cells, Mock-T cells, or PBS. Bioluminescence imaging and survival analysis revealed no significant differences in tumor dissemination among the three groups, indicating that gp350-CAR-T cells did not impact EBV-negative tumor cells

To further investigate whether gp350-CAR-T cells are effective against EBV- tumor cells, we examined their potential to prevent the dissemination of EBV- SUDHL-4 cells. A total of 1.5 × 10^7 fluorescently labeled SUDHL-4 cells were resuspended in 200 μL of PBS and injected into NOD-SCID mice via the tail vein (Fig. 7A). The mice were randomly assigned to three groups: a blank group (n = 4), a MOCK-T group (n = 4), and a gp350-CAR-T group (n = 4). All other experimental conditions were consistent with those used for EBV + tumor cells.

Our findings indicated no significant differences in tumor dissemination among the three groups (Fig. 7B). Euthanasia was performed when mice exhibited signs of distress, such as piloerection and significant weight loss.

## Discussion

The primary goal of this study was to evaluate the anti-tumor efficacy of gp350-CAR-T cells in EBV + Burkitt lymphoma. Our findings demonstrate that gp350-CAR-T cells significantly inhibit the growth and dissemination of EBV + Burkitt lymphoma cells, leading to extended survival in treated mice. These results support our hypothesis that gp350-CAR-T cells effectively target EBV-infected cells expressing the gp350 antigen. As an EBV-specific antigen, gp350 is predominantly expressed during the lytic phase of the virus, making it a highly specific target for CAR-T cell therapy [11, 13]. This specificity is crucial for ensuring that the treatment selectively targets tumor cells while sparing healthy tissues. Consistent with previous studies, our research indicates that gp350-CAR-T cells hold significant therapeutic potential for EBV-positive malignancies [14, 15]. Furthermore, this study provides new experimental evidence for the application of gp350-targeted therapy specifically in Burkitt lymphoma, a relatively underexplored area in prior research.

A notable strength of this study is the high specificity observed in gp350-CAR-T cell targeting against EBV + Burkitt lymphoma cells. This specificity is essential, as non-specific cytotoxicity can lead to severe adverse effects in patients receiving CAR-T cell therapy. Our experiments revealed that gp350-CAR-T cells exerted little to no tumor-lysing effect on the EBV- SUDHL-4 cell line while demonstrating strong tumor-killing efficacy against EBV + Namalwa and RAJI cells. Such precision is vital for developing CAR-T therapies, particularly for hematologic malignancies where off-target effects can be life-threatening. Reducing the risk of off-target toxicity not only enhances patient safety, but also improves overall treatment efficacy by maximizing the therapeutic dose against tumor cells [16, 17].

In addition to confirming the specificity of gp350-CAR-T cells, we observed a potential dose–response relationship. The efficacy of gp350-CAR-T cells appeared to be closely linked to the E:T ratio, with increased cytotoxicity at higher ratios. At a 2:1 E:T ratio, gp350-CAR-T cells exhibited markedly greater cytotoxic effects compared to a 1:1 ratio, suggesting a dose-dependent trend. This observation aligns with previous studies that reported similar dose–response patterns in other EBV + tumor models [14]. Certainly, further studies with additional E:T rations are needed to characterize the dose-dependent effects of these CAR-T cells comprehensively and conclusively on tumor cells. Understanding this potential dose-efficacy relationship is critical for optimizing CAR-T cell therapy, as it helps determine the appropriate number of cells to administer in clinical settings to achieve maximum therapeutic benefit while minimizing potential toxicity. The observed trend suggests that careful control of CAR-T cell dosing will be crucial in clinical applications; administering too low a dose may result in suboptimal efficacy, allowing tumor cells to evade immune surveillance [18], while administering too high a dose could lead to adverse immune reactions or cytokine release syndrome (CRS), a potentially life-threatening complication associated with CAR-T therapies [19, 20]. Future research should focus on optimizing dosing regimens to strike a balance between efficacy and safety [19], with large-scale clinical trials necessary to establish ideal dosing protocols across diverse patient populations.

Beyond the 1:1 and 2:1 E:T ratios evaluated in this study, examining a broader range could offer a more thorough understanding of the dose-dependent effects of gp350-CAR-T cells. Investigating lower E:T ratios may help identify the minimum effective dose for tumor cell eradication, which is crucial for establishing the threshold of CAR-T cell therapy efficacy. Moreover, exploring a wider range of E:T ratios could shed light on the capacity of gp350-CAR-T cells to address tumor heterogeneity and resistance mechanisms [21]. For example, tumors with high antigen density may respond effectively to lower E:T ratios, whereas those with low antigen expression or a suppressive microenvironment might require higher ratios for optimal targeting. Grasping these complexities is essential for personalizing CAR-T cell therapy to meet the unique needs of individual patients.

Compared to traditional chemotherapy, CAR-T cell therapy offers several advantages in terms of precision and reduced toxicity. While chemotherapy effectively kills rapidly dividing cells, it often causes collateral damage to healthy tissues, resulting in severe side effects such as immunosuppression, organ toxicity, and secondary malignancies [22, 23]. In contrast, CAR-T cell therapy is designed to specifically target tumor cells by recognizing surface antigens unique to those tumors, thus sparing normal cells [6]. Our study demonstrated that gp350-CAR-T cells specifically targeted EBV-positive tumor cells. Off-target effect experiments will be conducted subsequently. This precision not only improves patient outcomes but also enhances the overall quality of life for patients undergoing treatment by minimizing side effects compared to traditional therapies.

Furthermore, we observed that gp350-CAR-T cells secreted large amounts of IFN-γ and TNF-α when co-cultured with EBV + tumor cells, indicating their ability to trigger a robust anti-tumor immune response. These cytokines play critical roles in immune activation and tumor cell destruction [24–26]; IFN-γ enhances the cytotoxic activity of T cells and natural killer (NK) cells [24], while TNF-α promotes inflammation and apoptosis of tumor cells [25, 26]. The elevated levels of these cytokines observed in our study further validate the potential of gp350-CAR-T cells to induce a strong and sustained anti-tumor response in EBV-associated lymphomas. Gp350-CAR-T therapy provides renewed hope for patients with relapsed or refractory EBV + lymphomas. Early initiation of CAR-T cell therapy may lead to improved outcomes for certain newly diagnosed high-risk EBV + lymphomas patients. The ability of CAR-T cells not only to directly kill tumor cells but also to modulate the immune microenvironment enhances their therapeutic advantages over conventional treatments. Furthermore, it remains essential to investigate and validate whether gp350-CAR-T cells can effectively eliminate EBV and improve survival outcomes in patients with hemophagocytic syndrome induced by EBV infection.

While most studies have focused on CD19-targeted CAR-T cells for treating B-cell malignancies [27, 28], our research explores a novel therapeutic strategy by targeting the gp350 antigen in EBV + tumors. Previous research has established the efficacy of CD19 CAR-T cells in treating various B-cell malignancies such as acute lymphoblastic leukemia (ALL) and DLBCL [27, 29]. However, EBV-associated malignancies represent a unique subset driven by viral infection, where traditional CD19-targeted therapies may not be effective against all EBV-positive tumors. Our study provides new insights into the application of gp350-targeted CAR-T cells for EBV-positive Burkitt lymphoma, offering a promising alternative for patients who may not respond to CD19-based therapies. Moreover, the gp350 target identified in this study offers critical insights for designing multi-target, multi-epitope CAR-T products to address other EBV-associated malignancies [30].

Other studies have also demonstrated the potential of gp350-targeted CAR-T cells across various EBV-associated cancers; for instance, Zhang et al. reported strong anti-tumor effects of gp350-CAR-T cells in nasopharyngeal carcinoma (NPC), an EBV + epithelial malignancy [14]. These findings suggest that gp350 may serve as a broadly applicable target for CAR-T cell therapy across different EBV-driven cancers, expanding its potential clinical utility beyond Burkitt lymphoma and potentially benefiting a larger population of patients with EBV-associated malignancies.

Despite these promising results, our study has several limitations. Our experiments were primarily conducted using mouse models. While gp350-CAR-T cells demonstrated significant anti-tumor effects in these models, it remains uncertain how these results will translate to human patients due to differences in immune systems and tumor microenvironments between species. Future studies should utilize additional animal models or patient-derived xenograft (PDX) models to better simulate human immune responses and tumor biology. Another limitation is the lack of long-term safety data on gp350-CAR-T cells; further long-term studies are needed to assess potential risks such as immune toxicity, tumor relapse, and antigen escape—whereby tumor cells downregulate or lose the targeted antigen to evade immune detection [31, 32]. Future research should explore combination therapies involving gp350-CAR-T cells alongside immune checkpoint inhibitors like PD-1/PD-L1 inhibitors [33, 34] to enhance CAR-T cell persistence and prevent tumor evasion [35–37]. So the efficacy of CAR-T cells after infusion, as well as during disease progression or relapse, remains limited due to factors such as antigen escape, reduced CAR-T cell persistence, and the complex tumor microenvironment, highlighting the need for further improvements.Additionally, the complexity of CAR-T cell manufacturing, combined with the need for individualized dosing regimens and administration schedules to address the variability among patients and disease types, presents significant challenges for its clinical applications [21].

## Conclusion

In conclusion, our study provides strong experimental evidence supporting the use of gp350-targeted CAR-T cells for treating EBV + Burkitt lymphoma. By specifically targeting the gp350 antigen, these CAR-T cells can effectively eliminate EBV-infected tumor cells while minimizing damage to normal tissues. Although challenges remain—such as overcoming antigen escape and optimizing dosing strategies—this study lays a solid foundation for the clinical application of gp350-CAR-T cells. Future research should focus on expanding this approach’s therapeutic potential to other EBV-associated cancers while improving manufacturing processes and exploring combination therapies to maximize treatment efficacy and safety.

## Supplementary Information

Supplementary Material 1. Figure S1 Schematic diagram of the anti-gp350 CAR structure. The CAR construct includes: IgHL (Immunoglobulin heavy chain leader signal sequence), scFv (single-chain variable fragment for specific antigen recognition), IgG4 hinge (for receptor flexibility), IgG1 Fc CH3 domain (for structure stabilization), CD28TM (CD28 transmembrane domain for membrane anchoring), CD28cyto (CD28 cytoplasmic domain for costimulatory signaling), and CD3ζ (CD3 zeta chain intracellular domain for primary T-cell activation signal). The restriction sites PmlI and BamHI are indicated.

Supplementary Material 2. Figure S2 gp350-Targeted CAR-T cells with high viability and transduction rates. (A) (F): In the FSC vs SSC scatter plot, the gated cell populations for analysis are shown. The population adjacent to SSC, which was not gated, represents shrunken cells or debris. The gated populations were 72.51% in the CAR-T group and 29.44% in the MOCK-T group. (B) (G): In the FVD-KO vs SSC scatter plot, the gated FVD-negative live cell populations were 92.73% in the CAR-T group and 92.89% in the MOCK-T group. (C) (H): The proportion of CD3-positive T cells was 97.86% in the CAR-T group and 93.79% in the MOCK-T group. (D): The MOCK-T group served as a negative control, with the gating percentage set at 1.73%. (I): Using panel D as the background control, the transduction rate of CAR-T cells was 41.68%. (E) (J): In the CAR-T group, the proportions of CD4+ and CD8+ T cells were 33.02% and 60.61%, respectively. In the MOCK-T group, the proportions of CD4+ and CD8+ T cells were 35.34% and 53.91%, respectively.

Supplementary Material 3. Figure S3 Histological and immunohistochemical analysis of tumor tissue in mice. (A): HE stains of tumor tissues reveals dense, irregular tumor cells with a high nucleus-to-cytoplasm ratio in the MOCK-T group, indicative of active proliferation. Immunohistochemical analysis shows elevated Ki-67 and BCL-2 expression in the MOCK-T group, supporting its high proliferative activity and anti-apoptotic properties. In contrast, the gp350-CAR-T group displays extensive necrosis and apoptosis, with loosely arranged cells and elevated Bax expression, indicating enhanced apoptosis. Additionally, increased CD3-positive T cell infiltration and reduced gp350 levels in the gp350-CAR-T group confirm its targeted anti-tumor activity. (B): Quantification of the immunohistochemical staining for Bax, BCL-2, Ki-67, CD3, and gp350. The MOCK-T group exhibits significantly higher Ki-67 and BCL-2 levels, associated with greater proliferative activity and anti-apoptotic properties, while the gp350-CAR-T group shows markedly increased Bax expression, indicating enhanced apoptosis. CD3-positive T cell infiltration and reduced gp350 expression in the gp350-CAR-T group further confirm the specific anti-tumor effect of gp350-CAR-T cells.

## Data Availability

The data that support the findings of this study are available from the corresponding author (ZM.Z) upon reasonable request.
