# Supplementary figures and images for: Gp350-targeted CAR-T therapy in EBV-positive Burkitt lymphoma: pre-clinical development of gp350 CAR-T
Source: J Transl Med. 2025 Feb 10;23:171. doi: 10.1186/s12967-025-06188-w (PMC11809011; doi:10.1186/s12967-025-06188-w)

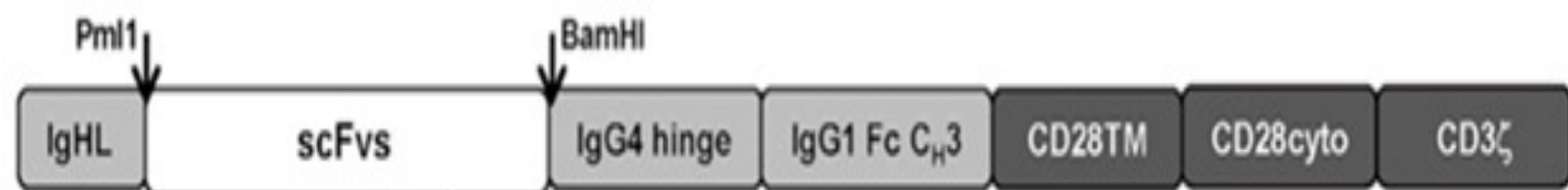

Supplement: Supplementary file 1 — Supplementary Material 1. Figure S1 Schematic diagram of the anti-gp350 CAR structure. The CAR construct includes: IgHL (Immunoglobulin heavy chain leader signal sequence), scFv (single-chain variable fragment for specific antigen recognition), IgG4 hinge (for receptor flexibility), IgG1 Fc CH3 domain (for structure stabilization), CD28TM (CD28 transmembrane domain for membrane anchoring), CD28cyto (CD28 cytoplasmic domain for costimulatory signaling), and CD3ζ (CD3 zeta chain intracellular domain for primary T-cell activation signal). The restriction sites PmlI and BamHI are indicated. [file 12967_2025_6188_MOESM1_ESM.pdf]

MOCK-T

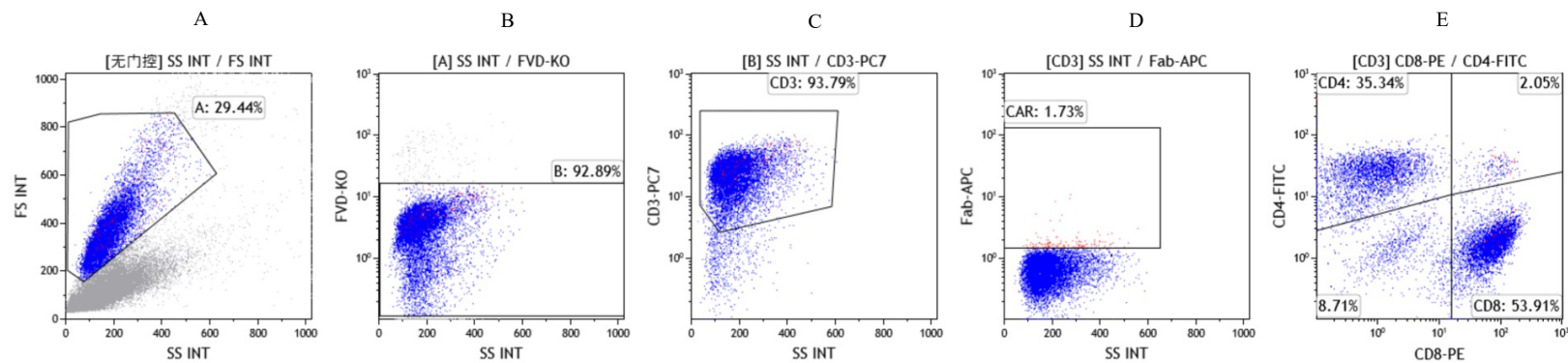

CAR-T

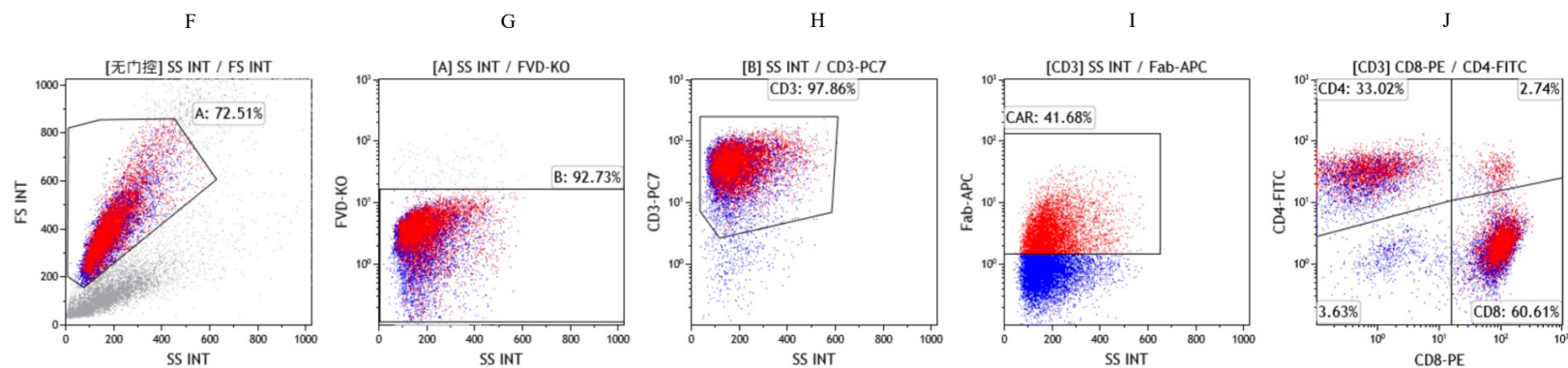

Supplement: Supplementary file 2 — Supplementary Material 2. Figure S2 gp350-Targeted CAR-T cells with high viability and transduction rates. (A) (F): In the FSC vs SSC scatter plot, the gated cell populations for analysis are shown. The population adjacent to SSC, which was not gated, represents shrunken cells or debris. The gated populations were 72.51% in the CAR-T group and 29.44% in the MOCK-T group. (B) (G): In the FVD-KO vs SSC scatter plot, the gated FVD-negative live cell populations were 92.73% in the CAR-T group and 92.89% in the MOCK-T group. (C) (H): The proportion of CD3-positive T cells was 97.86% in the CAR-T group and 93.79% in the MOCK-T group. (D): The MOCK-T group served as a negative control, with the gating percentage set at 1.73%. (I): Using panel D as the background control, the transduction rate of CAR-T cells was 41.68%. (E) (J): In the CAR-T group, the proportions of CD4+ and CD8+ T cells were 33.02% and 60.61%, respectively. In the MOCK-T group, the proportions of CD4+ and CD8+ T cells were 35.34% and 53.91%, respectively. [file 12967_2025_6188_MOESM2_ESM.pdf]

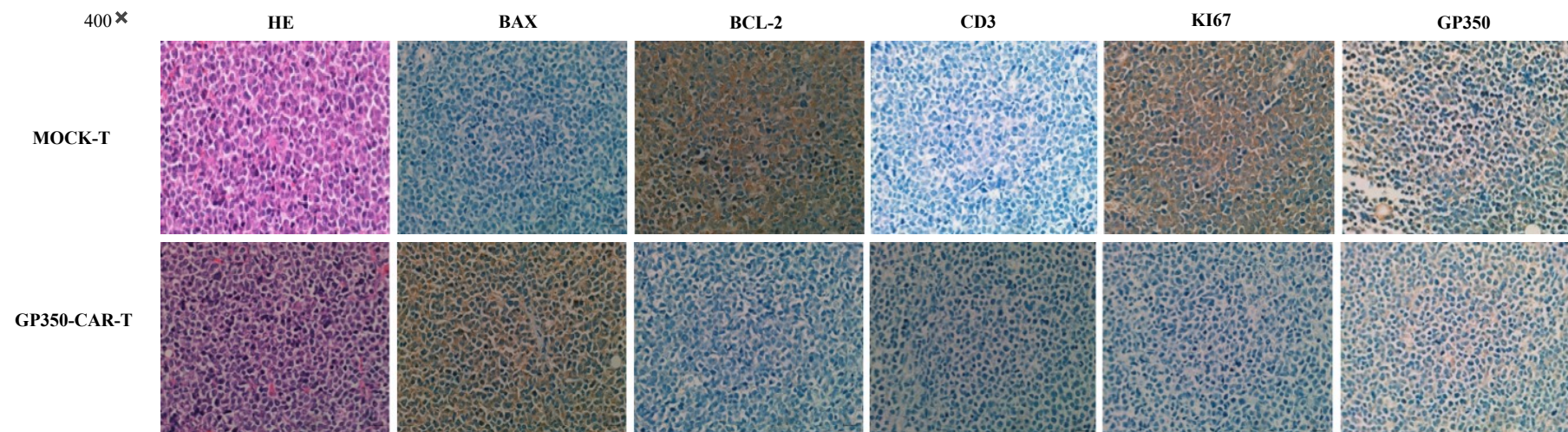

A

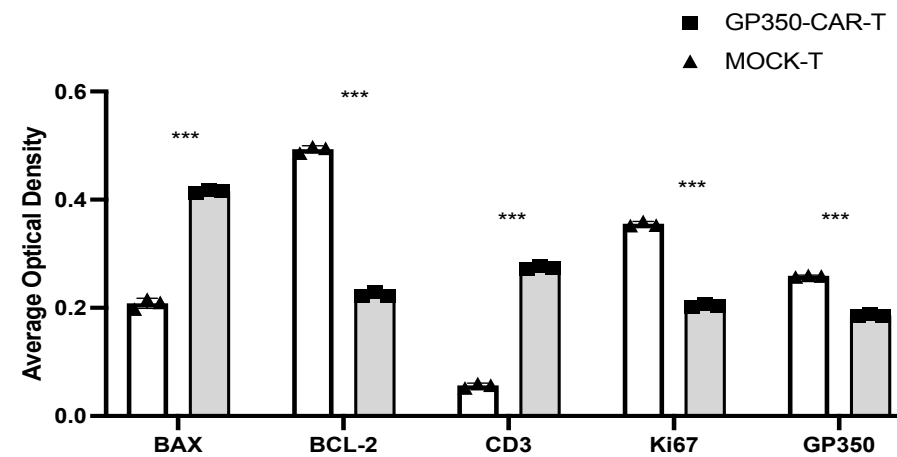

B

Supplement: Supplementary file 3 — Supplementary Material 3. Figure S3 Histological and immunohistochemical analysis of tumor tissue in mice. (A): HE stains of tumor tissues reveals dense, irregular tumor cells with a high nucleus-to-cytoplasm ratio in the MOCK-T group, indicative of active proliferation. Immunohistochemical analysis shows elevated Ki-67 and BCL-2 expression in the MOCK-T group, supporting its high proliferative activity and anti-apoptotic properties. In contrast, the gp350-CAR-T group displays extensive necrosis and apoptosis, with loosely arranged cells and elevated Bax expression, indicating enhanced apoptosis. Additionally, increased CD3-positive T cell infiltration and reduced gp350 levels in the gp350-CAR-T group confirm its targeted anti-tumor activity. (B): Quantification of the immunohistochemical staining for Bax, BCL-2, Ki-67, CD3, and gp350. The MOCK-T group exhibits significantly higher Ki-67 and BCL-2 levels, associated with greater proliferative activity and anti-apoptotic properties, while the gp350-CAR-T group shows markedly increased Bax expression, indicating enhanced apoptosis. CD3-positive T cell infiltration and reduced gp350 expression in the gp350-CAR-T group further confirm the specific anti-tumor effect of gp350-CAR-T cells. [file 12967_2025_6188_MOESM3_ESM.pdf]
